# Neural mechanisms supporting evaluation of others’ errors in real-life like conditions

**DOI:** 10.1038/srep18714

**Published:** 2016-01-05

**Authors:** Iiro P. Jääskeläinen, Hanna-Leena Halme, Yigal Agam, Enrico Glerean, Juha M Lahnakoski, Mikko Sams, Karoliina Tapani, Jyrki Ahveninen, Dara S. Manoach

**Affiliations:** 1 Brain and Mind Laboratory, Department of Neuroscience and Biomedical Engineering, Aalto University School of Science, FIN-00076, Espoo, Finland; 2 Department of Psychiatry, Massachusetts General Hospital, Harvard Medical School, Boston, MA 02114, USA; 3 Massachusetts General Hospital Athinoula A. Martinos Center for Biomedical Imaging, Harvard Medical School, Charlestown, MA 02129, USA; 4 Advanced Magnetic Imaging Centre, Aalto Neuroimaging, Aalto University, FIN-00076, Espoo, Finland

## Abstract

The ability to evaluate others’ errors makes it possible to learn from their mistakes without the need for first-hand trial-and-error experiences. Here, we compared functional magnetic resonance imaging activation to self-committed errors during a computer game to a variety of errors committed by others during movie clips (*e.g.*, figure skaters falling down and persons behaving inappropriately). While viewing errors by others there was activation in lateral and medial temporal lobe structures, posterior cingulate cortex, precuneus, and medial prefrontal cortex possibly reflecting simulation and storing for future use alternative action sequences that could have led to successful behaviors. During both self- and other-committed errors activation was seen in the striatum, temporoparietal junction, and inferior frontal gyrus. These areas may be components of a generic error processing mechanism. The ecological validity of the stimuli seemed to matter, since we largely failed to see activations when subjects observed errors by another player in the computer game, as opposed to observing errors in the rich real-life like human behaviors depicted in the movie clips.

Learning from errors is as fundamental to adaptive human behavior as learning from successful actions. It requires detecting errors, evaluating what went wrong, and adjusting behavior accordingly. Importantly, observing errors made by others makes it possible to learn from their mistakes without the need for first-hand trial-and-error experiences. One of the challenges to learning from errors by others is that they come in a great variety: inappropriate social behaviors, failed action sequences in sports performance, and falling down due to failing to notice that the ground is slippery are but few examples of the breadth of errors in everyday life.

Previous neuroimaging studies have largely focused on elucidating the neural responses to self-committed errors, which are characterized by increased hemodynamic activity in the anterior cingulate cortex (ACC) relative to correct responses^1^,^2^,^3^,^4^,^5^,^6^,^7^,^8^,^9^,^10^. Error-related ACC activation has been observed across a variety of experimental tasks and hence has been suggested to reflect the operation of a ‘generic’ error processing mechanism^11^ (note however that due to inherent limitations in neuroimaging methods, these tasks have been limited to relatively simple ones and thus, e.g., error responses during failures in social interactions or sports performance have not been studied). ACC has been further divided into dorsal and rostral subregions (dACC/rACC) with dACC activation suggested to reflect detection of errors and error-based reinforcement learning^12^ and rACC (and possibly also insula and amygdala^13^,^14^) the motivational or emotional significance of errors^3^,^8^,^10^. In addition to ACC, striatal dopaminergic mechanisms have been associated with error-based reinforcement learning during self-committed errors^11^,^12^.

The neural mechanisms supporting learning from observing errors committed by others have remained less extensively studied. One of the central research questions has been to what extent the neural mechanisms that process one’s own errors overlap with those that subserve learning from the errors of others. The concept that a set of neurons can play a role both in monitoring one’s own actions and those of others stems from the discovery of ‘mirror neurons’, *i.e.*, motor-cortical neurons that fire both when an animal executes an action and perceives similar actions performed by others^15^. Supporting such “mirroring” hypothesis, previous studies demonstrate that observing errors by another person engaged in non-naturalistic experimental tasks produces a similar but lower amplitude error-related negativity (ERN: an event-related potential that peaks ~100 ms following an error, initially termed N_E_^16^,^17^) as self-committed errors^18^,^19^. Further, ACC seems to be activated under both conditions^20^,^21^,^22^. In another study, however, error-related striatal responses were observed during active first-person play of a tank-shooting computer game and not during passive viewing of a record of game play^23^, thus suggesting differences between neural mechanisms that support processing of one’s own errors and those committed by others.

One can speculate that the smaller and/or lack of common error responses during other- vs. self-committed errors reflected that the tasks were too artificial to engage error processing . It is now possible to address this question by using movies as stimuli during functional magnetic resonance imaging^24^,^25^,^26^,^27^,^28^,^29^,^30^,^31^,^32^,^33^,^34^,^35^. This allows the presentation of errors by others in highly naturalistic and captivating stimulus conditions. Under these more naturalistic stimulus conditions we hypothesized that error processing mechanisms will be more robustly activated. We also addressed the question of whether a variety of naturalistic errors such as inappropriate social behaviors, failed sports performances, etc. are processed by the same brain areas as self-committed errors in computer games. This would support the hypothesis of a generic error-processing mechanism. Further, since observing errors requires simulation of correct behaviors and detection of deviations from them, it can be hypothesized that additional areas are recruited when observing errors in, e.g., social behaviors under natural viewing conditions.

Here, we presented in Experiment 1 healthy volunteers with short movie clips containing a variety of real-life like human errors during fMRI. We also recorded hemodynamic responses to self-committed and observed errors in a relatively simple cartoon-based computer game (see Fig. 1). We specifically hypothesized that regions consistently activated during errors in a variety of experimental paradigms, such as striatum and ACC, would be activated both during self-committed and observed errors. Such a finding would suggest that these regions are components of a generic error processing system. We also hypothesized that watching a variety of errors by others in real-life like situations will elicit more robust and widespread activity than observing errors in the computer game. This would highlight the importance of using naturalistic stimuli such as movie clips when studying the neural basis of error-observation. Further, we hypothesized that observing naturalistic errors by others will recruit additional regions to support simulation of other’s behaviors, including posterior temporal areas that are involved in social perception^36^ and anterior and posterior midline structures that together with parahippocampal gyrus have been proposed to underlie generation of scenarios of alternative behavioral sequences^37^. Finally, to determine whether we could replicate our findings in an independent dataset, we re-analyzed our previously published fMRI dataset wherein healthy volunteers were presented with a re-edited drama movie with errors predominantly in social interactions. In this Experiment 2, we had a separate group of volunteers rate the errors that they observed in the drama movie and used those ratings in a general linear model analysis to reveal brain areas that were activated during the errors. We hypothesized that similar areas are activated in this second experiment as during observation of human errors in the short movie clips.

## Results

In Experiment 1, significant activation was observed in multiple brain areas (see Fig. 2, top panel; For a summary listing of activity cluster peaks across experiments and conditions, see Table 1) following self-committed errors in the computer game, as compared with successful trials. In contrast, there was significant activity in only right IFG for passively viewed errors compared with passive viewing of successful trials (see Fig. 2, second panel from top). In the third condition of Experiment 1, where the subjects passively viewed errors committed by others in the short movie clips, there were multiple brain regions that exhibited significant responses to errors (see Fig. 2 third panel from top). Brain regions showing significant activation only during free viewing of others’ errors included middle temporal gyri (MTG), superior temporal sulci (STS), lateral occipital cortex (LOC), temporal pole (TP), lingual gyrus (LG), posterior cingulate cortex (PCC), precuneus, parahippocampal cortex (PHC), and amygdala. Further, within areas activated both by self-committed and observed errors, the precise loci of activity partly differed: activity of ACC extended more rostrally and more ventrally into medial prefrontal cortex (MPFC) during natural viewing errors by others. Further, hemodynamic activity observed in the putamen and globus pallidus was less widespread, and more posterior, during natural viewing of errors than in case of self-committed errors.

In Experiment 2, we tested how ratings of error observation explained brain hemodynamic activity during free viewing of a re-edited drama movie that contained errors mainly of the type of inappropriate social behaviors and misunderstandings of other’s intentions by the movie characters. This offered an independent replication of the natural-viewing condition in Experiment 1. The areas activated during errors in the drama movie are shown in the bottom panel of Fig. 2. There were some differences between the two natural-viewing conditions, with left IFG and premotor cortex (PMC), and superior frontal gyrus (SFG) significantly activated only in Experiment 2. Further, MPFC activity was seen more dorsally, rACC activation was lacking, and activity was more widespread on lateral surfaces of the temporal lobes and striatal structures in Experiment 2.

To determine which brain areas were specifically involved in processing errors in the different task/stimulus conditions of Experiment 1, we contrasted the hemodynamic responses between the conditions. As seen in the top panel of Fig. 3, self-committed errors elicited significantly stronger hemodynamic responses than observing errors committed by another player in the computer game in MFG, PMC, precentral gyrus/primary motor cortex (MI), insula, IFG, post-central gyrus, posterior parietal cortex (PPC), SMG, visual cortex, LG, cuneus, left precuneus, as well as thalamus, caudate nucleus, putamen, and globus pallidus bilaterally.

As seen in the middle panel of Fig. 3, errors committed by another player in the computer game elicited stronger responses than during observation of errors in the short video clips in right inferior frontal gyrus and left-hemisphere PPC. Errors committed by others in the short video clips elicited stronger activity in rACC, MPFC, orbito-frontal cortex (OFC), PCC, precuneus, and LG bilaterally, and in left-hemisphere PHC/HC and amygdala, as contrasted with observing another’s errors in the computer game. In addition, errors observed in the video clips elicited significantly stronger hemodynamic responses subcortically in left cerebellar culmen and caudate nucleus.

Finally, as seen in the bottom panel of Fig. 3, self-committed errors, contrasted with observation of errors by others in the short videoclips, activated the dorsolateral prefrontal cortex (DLPFC), IFG, precentral gyrus, PMC, insula, supramarginal gyrus (SMG), PPC, LOC, dACC, SMA, precuneus, caudate nucleus, putamen and globus pallidus bilaterally, as well as left substantia nigra and right claustrum. Observing errors by others in the videoclips, in turn, elicited stronger responses than self-committed errors in PCC, precuneus, superior and middle frontal gyri on the medial wall of the hemispheres, as well as in the right SMG, inferior parietal lobule (IPL), and angular gyrus.

Two control analyses were carried out for the natural-viewing condition in Experiment 1. First, it was determined which areas responded to surprising errors and which areas responded when errors seen in the movie clips were anticipated. As can be seen in Fig. 4, anticipated errors activated posterior parietal cortex, visual areas, and PHC. MPFC and rACC responded specifically to surprising errors. In the second control analysis, we examined which areas were activated by errors observed during natural viewing when observed errors where contrasted with (potentially confounding factor of) perceived pain in the videoclips. The results of this analysis are plotted in Fig. 5, and as can be seen, by comparing the activation maps in Fig. 2 (third panel from top) and those in Fig. 5, none of the error-related activations seemed to be confounded by effects of perceived pain to the characters. Only visual areas showed more widespread activity with effects of pain regressed out.

## Discussion

**Figure 1:**
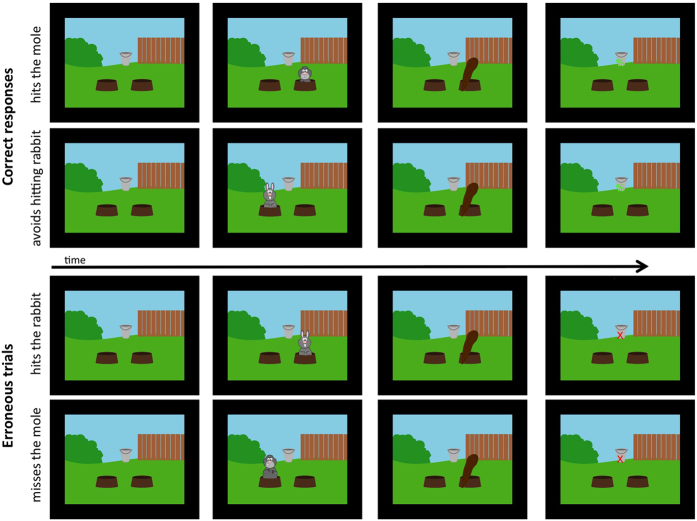
Correct and error trials in the whack-a-mole computer game that was used in Experiment 1. See text for details.

In the present study, we investigated whether errors committed by others activate the same generic error processing system as self-committed errors. We examined activation while subjects freely view a variety of errors committed by others in movie clips. These stimuli approximate the complexity and breadth of errors that take place in real life. To our knowledge, this has not been investigated previously. We also recorded brain responses to self-committed errors in a cartoon computer game (see Fig. 1) for a direct comparison of brain activity elicited during evaluation of errors by others and during self-committed errors.

**Figure 2:**
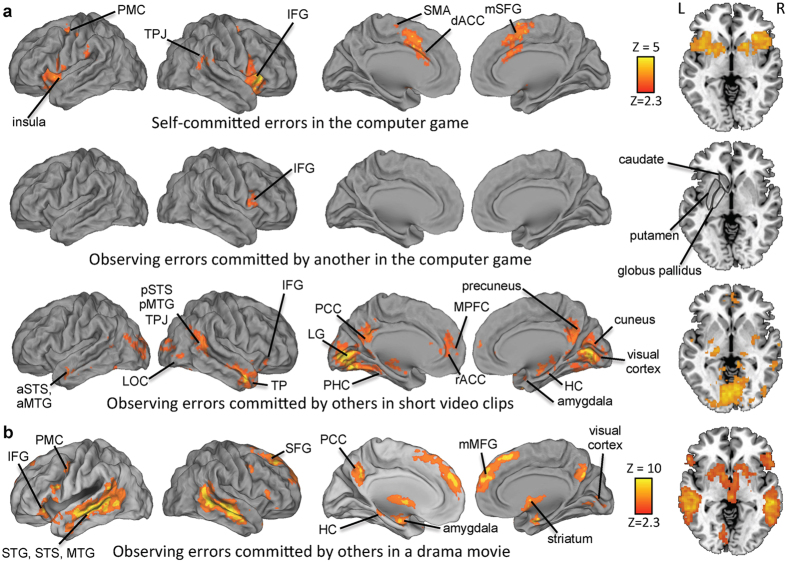
Brain regions showing statistically significant activity related to processing of self-committed and observed errors in Experiment 1 (topmost three panels) and observed errors in Experiment 2 (bottom panel) are indicated with the color maps overlaid on reconstructed cerebral hemispheres and on axial slice at z = −2 mm that cuts through the striatal structures. Shown are, starting from the top panel, brain regions that exhibited statistically significant responses following self-committed errors in the computer game, brain regions that exhibited statistically significant responses following observation of errors committed by another person in the computer game, brain regions with significant hemodynamic activity associated with observation of errors by others in the short video clips, and in the re-edited drama movie. All statistical parameteric maps were thresholded Z > 2.3 and cluster-wise corrected at P < 0.05.

**Figure 3:**
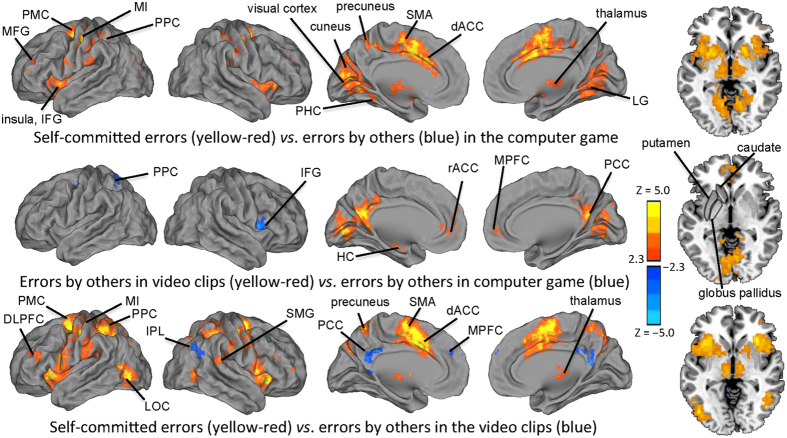
Brain regions wherein there were significant differences in hemodynamic activity between the conditions in Experiment 1 are indicated here with color maps overlaid on reconstructed cerebral hemispheres and on axial slice at z = −2 mm that cuts through the striatal structures. (TOP) The red-yellow color scale indicates brain regions that exhibited significantly larger hemodynamic responses following self-committed errors in the computer game than following watching of errors committed by another player. We failed to see any brain areas with significantly larger hemodynamic responses to errors committed by others in the game play as compared with responses to self-committed errors. (MIDDLE) The red-yellow color scale indicates brain regions that exhibited significantly larger hemodynamic responses following errors by others in the short video clips than following watching of errors committed by another player in the record of computer game play. The blue color indicates brain regions that exhibited significantly larger activity following observation of errors in the computer game play than following observation of the errors contained in the short video clips. (BOTTOM) The red-yellow color marks brain areas that exhibited significantly larger hemodynamic responses following self-committed errors in the computer game than following watching of errors committed by others in the short video clips. The blue color indicates brain regions that exhibited significantly larger activity following observation of errors by others in the video clips than following self-committed errors in the game play. All statistical parameteric maps were thresholded Z > 2.3 and cluster-wise corrected at P < 0.05.

Our findings, shown in Figs 2 and 3, revealed that there were activations in IFG and TPJ both during self-committed errors and during observation of errors by others in the videoclips and drama movie. In addition to these cortical areas, striatum showed significant activity both during natural viewing of mishaps by others and during self-committed errors in the computer game. Interestingly, we failed to see any striatal responses when the subjects perceived errors by another player in the computer game in Experiment 1. This suggests that natural viewing conditions are needed to study error processing in striatum, and might explain why some previous studies have also failed to document striatal responses to observed errors (23, although see also^20^). The striatal responses were especially robust when subjects viewed errors (of mostly social type) in the most naturalistic viewing condition, i.e., the drama movie in Experiment 2. Nonetheless, taken together, the present findings suggest that IFG, TPJ, and striatal structures contribute to a generic processing of both self-committed and observed errors.

It is naturally an intriguing question whether all these areas are specifically involved in error processing per se, or whether they were activated due to some other aspect common to the tasks. On this question, previous studies where processing of errors has been carefully teased apart from other information-processing steps offer possible interpretations. Specifically, IFG has been previously associated with detection of task-relevant cues^38^. These previous findings might explain why IFG was more robustly activated when subjects passively viewed game-play than during viewing of errors by others in the videoclips (see Fig. 3), given that the errors observed in the game contained salient task-related cues familiar to the subjects based on their own playing of the whack-a-mole game. Of note are also previous findings with non-naturalistic experimental paradigms in which some of the areas activated during natural viewing in the present study were associated specifically with error processing, including PCC^5^ and areas of MPFC close to rACC^5^ (for similar findings in non-human primates, see^39^). Thus, even though we failed to observe significant activations in PCC and MPFC during self-committed errors in the whack-a-mole game in the present study, the consistent results across the present natural-viewing conditions and previous studies with self-committed errors suggest that there is generic error processing in these areas.

In addition to PCC and MPFC, lateral and medial temporal lobe structures, LG, and IPL were activated specifically when observing errors committed by others in real-life like conditions (see Figs 2 and 3). The finding of IPL activation is similar to those in a previous study, where observing errors by others in a relatively simple go/nogo task were associated with error-observation specific responses in inferior parietal cortex^21^. Interestingly, the medial temporal lobe structures, MPFC, PCC, and precuneus together have been suggested to underlie generation of associations that form predictions, including mentally simulating scenarios and action sequences that can be subsequently retrieved upon encountering a situation where they can be useful in facilitating behavior^37^,^40^. Thus, it is possible that the events seen in the movies leading to errors were replayed/simulated by these brain structures after the errors to produce schemas of correct/successful actions. The across-experiments consistent activation of posterior-lateral temporal areas might, in turn, be related to recruitment of these areas in this process given their central role in processing of social cues^36^. Naturally, one has to keep in mind cautionary notes about dangers of reverse inference of assuming occurrence of specific cognitive operations based on foci of observed brain activity^41^.

**Figure 4:**
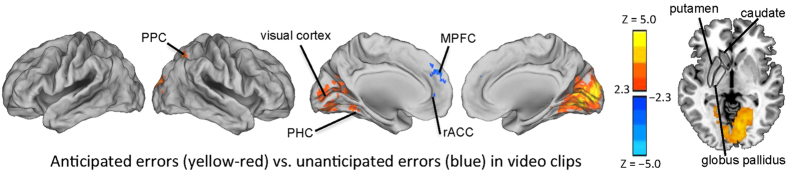
Brain regions showing significantly stronger activity during anticipated errors than surprising errors are shown with yellow-red color and brain areas showing stronger activity during surprising than anticipated errors are shown with blue color. Anticipated errors specifically activated PPC, visual cortex, and PHC. Surprising errors specifically activated MPFC and rACC. All statistical parameteric maps were thresholded Z > 2.3 and cluster-wise corrected at P < 0.05.

As another interesting finding, error-related activity was observed under natural viewing conditions in visual sensory cortical areas in both experiments (see Fig. 2). Tentatively, this might be related to previous findings of enhanced sensory cortical activity that took place after self-committed errors, possibly due to learning of sensory events that led to the error^42^. This hypothesis is supported by errors in the video clips that were not surprising to the subjects specifically eliciting responses in visual cortical areas (see Fig. 4). In contrast to this, significant MPFC and rACC activity was observed specifically after observing errors that were surprising to the subjects (see Fig. 4). MPFC activating during surprising events that violate predictions agrees well with findings from a previous study where both surprising errors and surprisingly successful actions elicited MFPC activity^22^. Significant rACC activation during natural viewing of human errors in Experiment 1 might, in turn, have been indicative of affective processing of the observed and surprising errors, given that rACC has been proposed to be activated during affective processing of self-committed errors^8^,^10^. Tentatively, lesser activity in dACC during free viewing of errors in the movie clips might have been due to participants not being proficient enough in, e.g., the athletic feats such as figure skating (in the context of which the errors took place) for them to model and learn from the errors that were shown. This possibility could be investigated in future studies.

In addition to similarities there were also some differences in the areas that were activated during perception of errors in the movie clips between the two experiments. This failure to see significant effect in both experiments for some of the brain areas could be explained by the fact that the type of human errors differed (*i.e.*, errors mostly in human action sequences in Experiment 1 *vs*. errors as inappropriate social behaviors and failures of the characters in reading the intentions of other characters in Experiment 2). Further, whereas the movie stimulus was presented with soundtrack in Experiment 2 the videoclips in Experiment 1 were silent, which might have also played a role, e.g., in eliciting more extensive responses in lateral-superior temporal areas in Experiment 2 (see Fig. 2). It has to be also kept in mind that ratings of error observation were obtained from a set of subjects separate from those who underwent fMRI scans in Experiment 2. This might have reduced the sensitivity of Experiment 2 as compared with Experiment 1 where the same subjects provided the self-ratings and were scanned with fMRI. On the other hand, it has been pointed out that the type of approach used in Experiment 2 is more conservative^43^. Nonetheless, we argue that the activations that replicated across the two natural viewing experiments (and which have been associated with processing of self-committed errors, either in the present or previous studies) are especially robust and reliable. Further, given that the breadth of errors observed was wider than in any previous study that we are aware of, our findings can be taken as providing rather robust support for the hypothesis that there are generic error processing mechanisms in the human brain.

**Figure 5:**
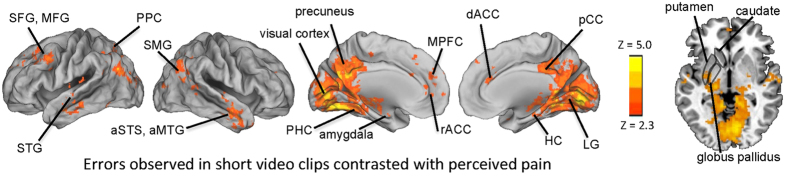
Brain regions showing significantly stronger activity during observed errors than during perceived pain in videoclips in Experiment 1. The areas significantly activated in this control analysis are plotted with yellow-red color. All statistical parameteric maps were thresholded Z > 2.3 and cluster-wise corrected at P < 0.05.

One of the fundamental challenges when using natural viewing conditions is that one has to trade the ability to control every aspect of the stimulus for ecological validity. Here, we argue that ecological validity allowed us to engage the brain’s error processing circuitry more robustly than what happened during observation of errors taking place in the relatively simple whack-a-mole cartoon-game, as evidenced by robust responses in areas associated with processing of self-committed errors. However, it is important to bear in mind that it is possible (despite that we used a wide array of different videoclips with highly variable sensory stimulation going with the different errors in Experiment 1 and that we included Experiment 2 as a within-study independent replication) that some aspects of the stimuli might have correlated with occurrence of the errors, and might thus have influenced some of the activations. Whilst sensory aspects of the stimulus, such as certain colors or movements, would be expected show up as activations in the corresponding sensory areas (but would had been unlikely to have given rise to specific responses in higher-order areas such as MPFC), affective-cognitive factors, such as perception of pain (e.g., figure skater falling down painfully), co-occurring with some of the errors might have modulated activity in the areas such as MPFC and rACC. To control for this, we ran in Experiment 1 a separate analysis where error ratings were contrasted with ratings of perceived pain. As can be seen in Fig. 5, multiple areas, including rACC and MPFC, appeared to be unrelated to the pain perceived in the videoclips. We warmly recommend that studies using naturalistic stimuli in the future include these types of control analyses that help pinpoint specific effects of interest and circumvent the potential problems caused by trading of strict experimental control for ecological validity.

Taken together, our results disclose sets of brain structures that support evaluation of human errors under naturalistic viewing conditions. Brain areas specifically responding during evaluation of errors by others under natural viewing conditions included lateral and medial temporal lobe structures, PCC, precuneus, and aspects of MPFC, which might have indicated simulation and storing for future use of alternative action sequences that could have led to successful behaviors. IFG, TPJ, and striatum were activated both during observation of errors by others in movie clips and during self-committed errors in the cartoon computer game. Further, MPFC and PCC, that were activated during natural viewing of errors in the present study, have been in previous studies shown to be activated during self-committed errors. Of these regions, IFG has been previously associated with processing of task-relevant cues, which might explain why it was most robustly activated during playing and observation of the whack-a-mole game. The TPJ, MPFC, PCC, and striatum can be seen as associated with rather generic error processing mechanisms. Finally, ecological validity of the stimuli seemed to play a significant role, since we largely failed to see activations when subjects observed errors by another player in the cartoon computer game, as opposed to errors in the complex and rich real-life like human behaviors depicted in the movie clips.

## Methods

### Subjects

In Experiment 1 there were 22 healthy volunteers (ages 21–50 years, mean 26, 10 females) who participated in the fMRI and behavioral data collection. One subject was unable to complete the scan due to uncomfortable sensation inside the scanner and was excluded from the analysis. In Experiment 2, there were 17 healthy volunteers (ages 21–25 years; mean 23; 7 females) who participated in a behavioral experiment the results of which were utilized in a model-based analysis of our previously collected fMRI dataset^44^. In our previously published fMRI dataset, there were 16 healthy volunteers (2 left handed; ages 22–43 years; mean 28; 3 females). None of the subjects in any of the experiments had any neurological or psychiatric diseases or medications affecting the central nervous system. Permission for the research was acquired from the ethical committee of Aalto University, the research was carried out in accordance with the guidelines of the declaration of Helsinki, and written informed consent was obtained from each subject prior to participation.

### Tasks and Stimuli

Experiment 1 consisted of three tasks, presented in the same order for all subjects during fMRI without auditory stimuli. In the first task the subjects played a video game (Fig. 1) by pressing buttons with their left and right index fingers. The game scene included a background picture of a garden and two holes on both sides of the screen. Two cartoon characters, a rabbit and a mole, emerged from either hole one at a time in a pseudo-random order for 700 ms, and then disappeared back to the hole. The participants’ task was to whack the mole by pressing the button of the side where the mole appeared. If a rabbit appeared, they should avoid hitting it and instead whack the empty hole on the opposite side (note that in this sense the task was not a classical response-inhibition task, as substitute response was required). Whacking the rabbit and failing to whack the mole were considered errors, as well as not reacting within the 700-ms time window during which the animal was visible in each trial. Visual feedback (green “%” indicating success and red “X” indicating error) appeared on the screen after each trial and reaction times were recorded. The total duration of each trial was 2 s, consisting of the emergence and disappearance of the animal (up to the reaction time, maximum 700 ms), response during which the club action was displayed (200 ms), presentation of the feedback sign (500 ms), and null time up to the end of the 2 s trial duration. Inter-trial interval (ITI) was, with equal probability, either 0, 1, 2, 3, 4, or 5 s, the average ITI being 1.86 s. The total duration of the game was approximately 15 minutes.

In the second task of Experiment 1, the subjects passively watched a 15-minute screen-capture recording of the whack-a-mole game as played by another person. Recording only presented the game as it appeared on the screen, *i.e.*, the other player was not visible to the subjects. The task was to passively observe the recording. The recording included a total of 26 errors, pooled across “missing the mole” and “whacking the rabbit” error types.

In the third task of Experiment 1, the subjects watched short video clips depicting human errors in naturalistic conditions, such as a figure skater falling down in the middle of her performance and a person slipping and falling down on an icy street, but also including video clips presenting faux pas type of errors such as the comedy character Mr. Bean engaging in behaviors that violate social norms. Thus, the definition of an error in this paradigm was rather broad, as we defined errors generally as failures by the characters to behave in accordance with their perceived goals/intentions (e.g., figure skaters aiming for successful performance) and in accordance with social norms (e.g., the Mr. Bean clips). The video included 35 errors in total. Because we wanted to ensure that the subjects did not expect errors to occur in every video clip, video clips without any errors were also included in the set that was presented.

In Experiment 2, we analyzed our previously published fMRI dataset. Detailed description of experimental details of this fMRI study can be found in^44^; briefly: 23 minutes of a re-edited version of a drama film “Match Factory Girl” (1990, directed by Aki Kaurismäki) was shown to healthy subjects during 3-Tesla fMRI, and the task of the subjects was to watch the movie as if they would in a movie theater. The movie depicted erroneous human behaviors (mainly inappropriate social behaviors and misunderstandings of other’s intentions by the movie characters). To obtain estimates of perceived errors for the re-analysis of these fMRI data in the Experiment 2 of the present study, an independent sample of volunteers behaviorally rated the presence of erroneous behaviors taking place in the movie (for details, see below).

### MRI and fMRI data collection

In Experiment 1, fMRI scanning was conducted in the Advanced Magnetic Imaging (AMI) Centre of Aalto University School of Science and Technology. BOLD fMRI was performed with 3.0 T Siemens Magnetom Skyra MRI scanner (Siemens Healthcare, Erlangen, Germany) using a 30-channel head coil and a standard T2* weighted EPI sequence. The imaging area consisted of 32 whole-brain functional gradient-echo planar oblique slices (slice thickness 4.5 mm, in-plane resolution 3.5 mm × 3.5 mm, field of view 224, voxel matrix 64 × 64, TE 30 ms, TR 2 s, flip angle 75). The slices were acquired in ascending interleaved order. In addition, anatomical whole-brain T1 images with 176 oblique slices were acquired using a magnetization prepared rapid gradient-echo (MPRAGE) sequence (TR 2530 ms, TE 3.3 ms, TI 1100 ms, flip angle 7, slice thickness 1.0 mm, FOV 256). In Experiment 2, we analyzed our previously published fMRI dataset, wherein anatomical T1-weighted MRI data and fMRI data were obtained with a 3.0 T GE Signa Excite MRI scanner (GE Medical Systems, USA) using a quadrature 8-channel head coil. Briefly, fMRI data were obtained with the following parameters: 29 slices, slice thickness 4 mm, in-plane resolution 3.4 × 3.4 mm, TE 32 ms, TR 2 s, flip angle 90. For a detailed description of MR-acquisition parameters, see^44^.

### Collection of self-rating measures of error perception

In the naturalistic-viewing condition in Experiment 1, the subjects were replayed the clips after the fMRI scanning session outside of the scanner. In-house annotation software^34^ was used to collect ratings for the intensity of observed errors. In addition, for control analyses, ratings were collected for the intensity of error anticipation (some of the errors were surprising and others easy to foresee), and intensity of perceived pain in the video clips associated with some of the errors (e.g., a person in the videoclip slipping and falling painfully on icy surface). For each of these ratings, subjects watched the video clips on a computer screen and gave ratings continuously by moving the mouse up and down. Ratings were collected at 5-Hz on a continuous scale from 0 (no observed error/error anticipation/perceived pain) to 1 (very intense observed error/error anticipation/perceived pain). In Experiment 2, subjects were shown the re-edited version of the movie that was used as the stimulus in our previous fMRI study^33^. The same annotation tool^34^ was used to record self-ratings of error occurrence as in Experiment 1, i.e., the subjects watched the re-edited drama movie on a computer screen and gave ratings continuously by moving the mouse up and down, with ratings collected at 5-Hz on a continuous scale from 0 (no observed error) to 1 (very intense observed error).

### Data analyses

In both Experiment 1 and 2, FSL software was utilized to carry out standard preprocessing steps. For Experiment 1, the first three volumes of each run were discarded, slice timing was corrected with sinc interpolation, and head motion was corrected with MCFlirt. Runs with displacement over 2 mm were excluded: the final analyses included 18 subjects (game playing), 19 subjects (passive game watching) and 18 subjects (natural scene videos). Functional data were spatially smoothed with a 8-mm FWHM Gaussian filter and high-pass filtered with a cut-off of 0.01 Hz. The data were then co-registered to template by first registering EPI to structural volume with 9 degrees of freedom (DOF) and then anatomical images were co-registered to standard MNI (Montreal Neurological Institute) 152 2 mm template with 9 DOF. BOLD time-series were further pre-whitened with FMRIB’s Improved Linear Model (FILM) to minimize temporal autocorrelation. Finally, motion parameters were used to regress out motion artifacts. In Experiment 2, we re-analyzed our previously published fMRI data that were preprocessed as follows: the first 10 volumes of the session were excluded from the analysis, the data were motion corrected with MCFlirt and non-brain matter was removed using BET. Spatial smoothing was conducted with a Gaussian kernel of 6 mm (FWHM), and high-pass filtering with 100-s cutoff. The data were co-registered (FLIRT) first to anatomical image allowing 7 DOF and then to MNI152 standard space allowing 12 DOF.

In Experiment 1, GLM analysis was performed with FSL’s FMRI Expert Analysis Tool (FEAT; www.fmrib.ox.ac.uk). Finite-impulse response (FIR) estimates of the event-related hemodynamic responses were calculated for errors and correct trials in the active whack-a-mole game playing (whereupon motor responses should cancel each other out) and passive game observation tasks. The FIR model included five consecutive post-stimulus 2-sec time points. We did not use any orthogonalization of the regressors, since the stimuli were randomly jittered. The model was fitted voxel-wise using ordinary least squares estimation, thus yielding average signal intensity in five post-stimulus time points. Five parameter estimates were computed, corresponding to the significance of activity at each post-error time point. The contrasts of error *vs.* correct was calculated for self-committed and observed errors.

For the third task of Experiment 1 (*i.e.*, human errors in video clips), event-related design matrices were constructed using the individual dynamic self-ratings (see above) as parametric modulators of the regressor functions. The rating time series were first down-sampled from 5-Hz to match the TR of 2 s. The ratings were then normalized to Z-scores and averaged over subjects. The regressors were convolved with a FIR set that included five basis functions. Contrast images were constructed individually for each subject with time points that did not contain any errors serving as the baseline. In the group-level analysis, individual contrast estimates were subjected into a mixed-effects model using FMRIB’s Local Analysis of Mixed Effects to detect mean group effect. Z-statistic images were thresholded using clusters determined by Z > 2.3 and a corrected cluster significance threshold of p < 0.05, using Gaussian Random Field theory^45^; these thresholds were used in all contrasts, including contrasts of anticipated vs. surprising errors as well as observed errors vs. perceived pain.

In Experiment 2, the fMRI data analysis was carried out using SPM8 software (see www.fil.ion.ucl.ac.uk/spm/software/). The error-perception time courses were averaged across subjects, down-sampled to the rate of TR, and utilized as regressors in a general linear model based analysis. Regressors were convolved with the standard hemodynamic response function. The resulting statistical parametric maps were thresholded as in Experiment 1.

## Additional Information

**How to cite this article**: Jääskeläinen, I. P. *et al.* Neural mechanisms supporting evaluation of others' errors in real-life like conditions. *Sci. Rep.* **6**, 18714; doi: 10.1038/srep18714 (2016).

## Figures and Tables

**Table 1:** Activity cluster peak coordinates in the different experimental conditions.

Condition	Brain region	MNI coordinates			
		x	y	z	Max Z
Exp1: self-committed errors	Insula R	32	26	0	4.7
	Supplementary motor area R	6	14	56	4.6
	Insula L	−44	10	−4	4.1
	Superior temporal gyrus L	−66	−28	20	4.1
	Superior temporal gyrus R	60	−40	18	3.8
Exp1: observed errors (game)	Inferior frontal gyrus, opercular, R	46	14	8	3.5
Exp1: observed errors (videos)	Lingual gyrus L	−12	−74	−12	4.9
	Inferior temporal gyrus R	52	8	−32	4.4
	Middle temporal gyrus R	56	−42	4	3.8
	Middle temporal gyrus L	−54	2	−20	3.6
	Anterior cingulum R	0	48	24	3.6
Exp1: self committed > observed (game)	Putamen R	18	12	−6	5.0
	Middle cingulum R	6	2	46	4.8
	Putamen L	−20	8	−6	4.8
	Calcarine gyrus L	−2	−78	12	4.3
	Precentral gyrus L	−54	0	20	3.5
Exp1: observed > self committed (videos)	posterior cingulum L	−4	−42	32	−4.2
	anterior cingulum R	0	48	26	−3.9
	angular gyrus R	46	−64	40	−3.5
Exp1: observed (videos) > observed (game)	calcarine gyrus R	0	−62	22	5.1
	medial superior frontal gyrus R	2	60	26	3.6
	middle frontal gyrus L	−18	26	38	3.5
	hippocampus L	−24	−14	−18	3.3
	anterior cingulum R	8	46	0	3.1
Exp1: observed (game) > observed (videos)	superior parietal lobule L	−24	−56	66	−4.4
	opercular inferior frontal gyrus R	56	12	8	−4.0
	precentral gyrus L	−28	−4	44	−3.5
Exp2: errors observed in drama film	orbitofrontal cortex L	−50	24	−8	13.3
	middle temporal gyrus R	52	−20	−8	11.8
	frontal angular area R	14	6	58	11.6
	amygdala R	24	−8	−20	9.5
	precuneus	0	−64	34	8.9
	vermis cerebelli IX	0	−62	−48	8.7
	precentral gyrus R	48	4	44	7.2
	crus cerebelli II L	−22	−86	−48	6.9
	prestriate cortex R	14	−80	0	5.9
	precentral gyrus L	−46	2	42	5.5
